# Inhibition of GABA_A_R or Application of *Lactobacillus casei* Zhang Alleviates Ulcerative Colitis in Mice: GABA_A_R as a Potential Target for Intestinal Epithelial Renewal and Repair

**DOI:** 10.3390/ijms231911210

**Published:** 2022-09-23

**Authors:** Qiuzhen Wang, Ziteng Deng, Jing Lan, Dan Li, Kai Fan, Jianyu Chang, Yunfei Ma

**Affiliations:** College of Veterinary Medicine, China Agricultural University, Beijing 100193, China

**Keywords:** ulcerative colitis, GABA_A_R, *Lactobacillus casei*, behavior, microbiota

## Abstract

Emerging evidence indicates that the gamma−aminobutyric acid type A receptor (GABA_A_R) and *Lactobacillus casei* Zhang regulate colitis in a variety of ways, such as by participating in host immune and inflammatory responses, altering the gut microbiota, and influencing intestinal barrier function. However, not much is known about the mechanisms by which GABA_A_R and *L. casei* affect colon epithelial cell renewal and the interaction between GABA_A_R and *L. casei* during this process. To elucidate this, we established a dextran sulfate sodium (DSS)−induced model and measured the mouse body weights, colon length, the disease activity index (DAI), and histological scores. Our results indicated that inhibition of GABA_A_R alleviated the DSS−induced colitis symptoms, resulting in less weight loss and more intact colon tissue. Moreover, treatment with bicuculline (Bic, a GABA_A_R inhibitor) increased the levels of PCNA, β−catenin, and TCF4 in mice with colitis. Interestingly, open field test performances showed that inhibition of GABA_A_R also attenuated colitis−related anxiety−like behavior. By 16S RNA gene sequencing analysis, we showed that inhibition of GABA_A_R partially reversed the gut dysbacteriosis of DSS−induced mice and increased the abundance of beneficial bacteria. Additionally, *L. casei* Zhang supplementation inhibited the expression of GABA_A_R in mice with colitis, promoted the proliferation and renewal of colon epithelial cells, and alleviated anxiety−like behavior and intestinal microflora disorder in mice. Thus, GABA_A_R plays a key role in the beneficial effects of *L. casei* on DSS−induced colitis in mice.

## 1. Introduction

Ulcerative colitis (UC) is an inflammatory bowel disease (IBD) that results in abdominal pain, diarrhea, and blood and mucus in the stool, as well as an increased propensity for malignancy [1]. It is characterized by the upregulation of proinflammatory cytokines, apoptotic signals, and an imbalance between microbiota and mucosal immunity [2]. The etiology and pathogenesis of UC are complicated, including genetic factors, microbiota, dysregulation of immune responses, and dysfunction of the mucosal barrier [3]. Even though it is widely accepted that dysfunction of the intestinal barrier plays a key pathophysiological role in UC, the specific causes of UC are not completely identified [4]. As a protective interface in the gastrointestinal (GI) tract, the colonic epithelium is not only a physical barrier separating the microbes from the host but also a key physiological feature in maintaining immune homeostasis [5]. Colonic epithelial injury represents a major physiological challenge that requires the rapid renewal and continuous replenishment of stem cells present in the niche at the base of the crypt [6]. Multiple pathways including the Notch, Hippo/Yes−associated protein, and Wnt/β−catenin pathways are involved in the epithelial regeneration process [7]. The Wnt/β−catenin signaling pathway is essential in stem cell proliferation and differentiation capacity. β−catenin is a key component of the Wnt/β−catenin signaling pathway. Upon activation, β−catenin is separated from the β−catenin destruction complex comprising APC, Axin, and GSK−3β, accumulated, and transferred to the nucleus, where it binds to TCF/LEF to activate target genes and promote the proliferation of intestinal stems cells [8]. In the present study, we mainly focused on the effect of the Wnt/β−catenin pathway on colonic epithelial proliferation and renewal in DSS−induced colitis in mice.

Gamma−aminobutyric acid (GABA) is mainly known as a major inhibitory neurotransmitter in the central nervous system. GABA is produced from glutamate through decarboxylation by glutamic acid decarboxylase (GAD) and is widely found in relatively large amounts, regulating various physiological and psychological processes such as anxiety and depression [9,10]. The GABA type A receptor (GABA_A_R) is one of the main GABA receptors. Previous studies have shown that GAD, GABA, and GABA_A_R are expressed in the small intestine and colon and that the GABA−GABA_A_R system directly alters the excitability of enteric nervous system neurons, spontaneous colonic contractility, and GI motility [11,12,13]. Although it is well known that colonic epithelial cells are enriched with a functional GABAergic signaling system [12], relatively little is known about the function of GABA_A_R in epithelial tissue renewal.

Patients with UC more often experience mental symptoms such as depression and anxiety compared to the general population [14,15,16]. The underlying mechanism of UC−induced behavioral disorders is not completely understood. Data from recent years indicate that the gut microbiome is closely related to neuroinflammation and further emotional behavior disorders [17,18]. Probiotics can be utilized to modify the intestinal microbiota of the host and may have a therapeutic potential in IBD patients [19,20]. As an important probiotic genus, *Lactobacillus* have been widely used in clinical trials and showed a wide range of positive effects, including reducing fat mass accumulation and central obesity, maintaining the diversity of intestinal microbiota, and preventing stress−related abdominal symptoms. [21,22,23]. Although a growing body of evidence has shown a close connection between *Lactobacillus* and UC, very few studies have pinpointed the role of the GABA system in the regulatory effects of *Lactobacillus* in emotional behavior disorders. The present research aimed to investigate the protective effect of *Lactobacillus* in DSS−induced colitis in mice by evaluating its impact on colonic epithelial proliferation and associating this with its well−known microbiota regulatory properties, paying special attention to the role of GABA_A_R.

## 2. Results

### 2.1. GABAergic Signals Were Upregulated in the Colon of DSS−nduced Mice

**Figure 1:**
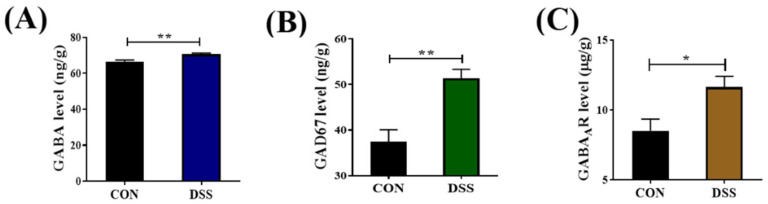
The expression levels of GABAergic signaling proteins were upregulated in the colon of ulcerative colitis (UC) mice. The levels of (**A**) GABA, (**B**) GAD67, and (**C**) GABA_A_R in the colon of DSS−induced mice were detected by ELISA kits. Data are presented as mean ± SEM (*n* = 5 per group). * *p* < 0.05, ** *p* < 0.01 vs. the control group.

To investigate the role of GABAergic signals in DSS−induced colitis, we first determined the expression levels of GABA, glutamic acid decarboxylase 67 (GAD67), and GABA_A_R in the colon by ELISA. GAD67 is the major enzyme converting glutamate into GABA. In comparison with the control group, the expression levels of GABA, GAD67, and GABA_A_R in the DSS group were significantly increased (*p* < 0.05), as shown in Figure 1.

### 2.2. Clinical Symptoms

**Figure 2:**
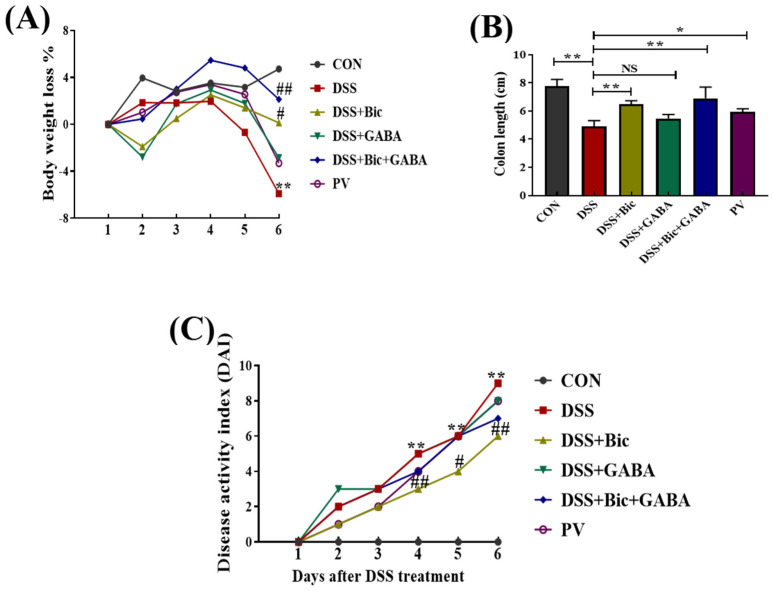

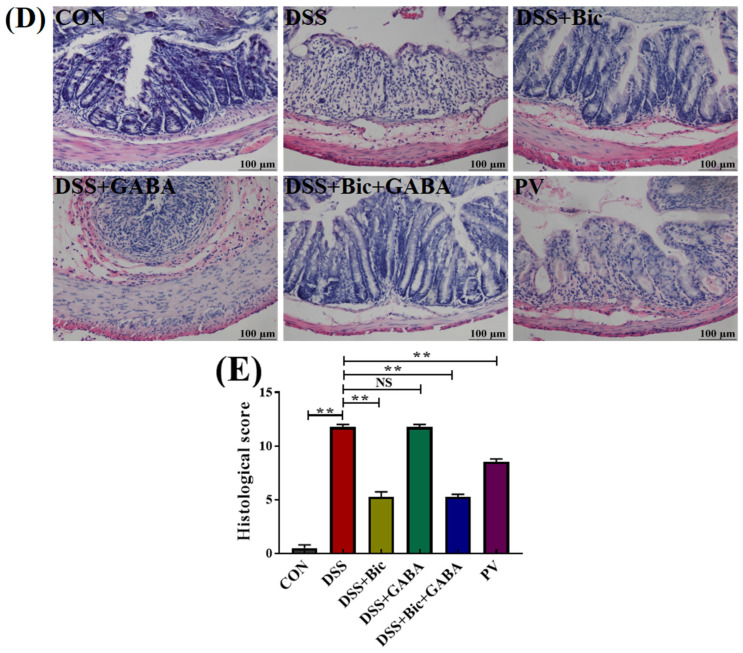
Effect of GABAergic signal and *L. casei* Zhang on DSS−induced colitis mice. Mice receiving 4% DSS in drinking water were treated with or without Bic and GABA during the six−day experiment. (**A**) Body weight loss. (**B**) Colon length. (**C**) DAI scores. (**D**) Representative H&E staining images of a colon. Scale bar = 100 μm. (**E**) Histological score. Data are presented as mean ± SEM (*n* = 10 per group). * *p* < 0.05, ** *p* < 0.01 vs. the control group; ^#^ *p* < 0.05, ^##^ *p* < 0.01 vs. the DSS group. NS indicates no significant change.

Based on the high expression levels of GABA_A_R, GAD67, and GABA in DSS−induced colitis mice and our previous studies on *Lactobacillus casei* Zhang [24], we sought to investigate the effects of the GABAergic system and *L. casei* Zhang on DSS−induced acute colitis in mice. As shown in Figure 2A,B, the DSS−induced model mice showed obvious clinical characteristics, such as weight loss, diarrhea, and blood stool, as indicated by a significantly increased disease activity index (DAI) score during 6 days of DSS treatment (*p* < 0.05). Mice treated with bicuculline (Bic, a GABA_A_R inhibitor) and *L. casei* Zhang showed significant improvements in weight loss and a reduction in DAI scores, indicating remission of colitis in mice. Further, we found that Bic, Bic + GABA, and *L. casei* Zhang treatment significantly increased colon length in DSS−induced mice (*p* < 0.05). Histological analysis was used to assess the severity of colonic inflammation in each group. Compared to control group, DSS−induced mice displayed severe pathological changes in their colons, including epithelial erosion, loss of crypts, destroyed muscularis mucosae, and immune cell infiltration. On the contrary, these symptoms were remarkably ameliorated by Bic, Bic + GABA, and *L. casei* Zhang treatments (*p* < 0.05, Figure 2D). The histopathology scores in the DSS + Bic, DSS + Bic + GABA, and *L. casei* Zhang prevention (PV) groups were significantly lower than those of the DSS group (*p* < 0.05). These results suggested that GABA_A_R inhibition could alleviate colitis in mice, and the therapeutic effect of *L. casei* Zhang on acute colitis was similar to that of GABA_A_R inhibition.

### 2.3. L. casei Zhang Reduced the Expression of GABA_A_R in Colitis

**Figure 3:**
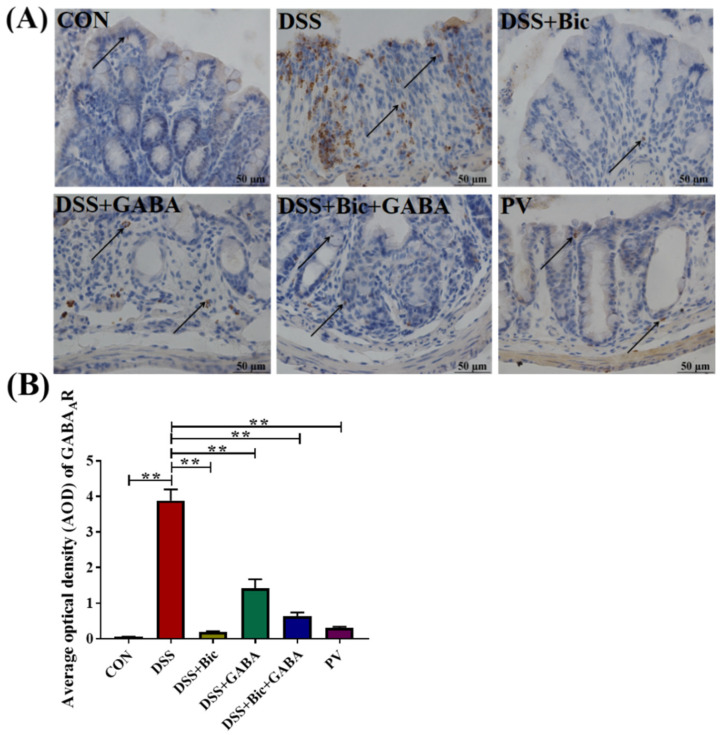
Evaluation of GABA_A_R expression in the mouse colon. (**A**) Representative immunohistochemistry data for GABA_A_R staining (brown) in the colon. Arrows point towards the GABA_A_R signal. (**B**) Statistical analysis of GABA_A_R expression. Data are presented as mean ± SEM (*n* = 5 per group). ** *p* < 0.01 vs. the control group.

In order to study the possible mechanism of the protective effect of *L. casei* Zhang, we analyzed GABA_A_R expression in the colonic segments in six groups. The expression of GABA_A_R was significantly increased in the DSS group compared with the control group (*p* < 0.05, Figure 3A,B). In addition, GABA_A_R was widely expressed in glandular epithelium and submucosa. Notably, the expression levels of GABA_A_R in the Bic, GABA, Bic + GABA, and *L. casei* Zhang treatment groups were much lower than that in the DSS group, demonstrating that *L. casei* Zhang could alleviate DSS−induced acute colitis by inhibiting the expression of GABA_A_R.

### 2.4. Inhibition of GABA_A_R Promotes Colonic Epithelial Renewal in Mice with Colitis

**Figure 4:**
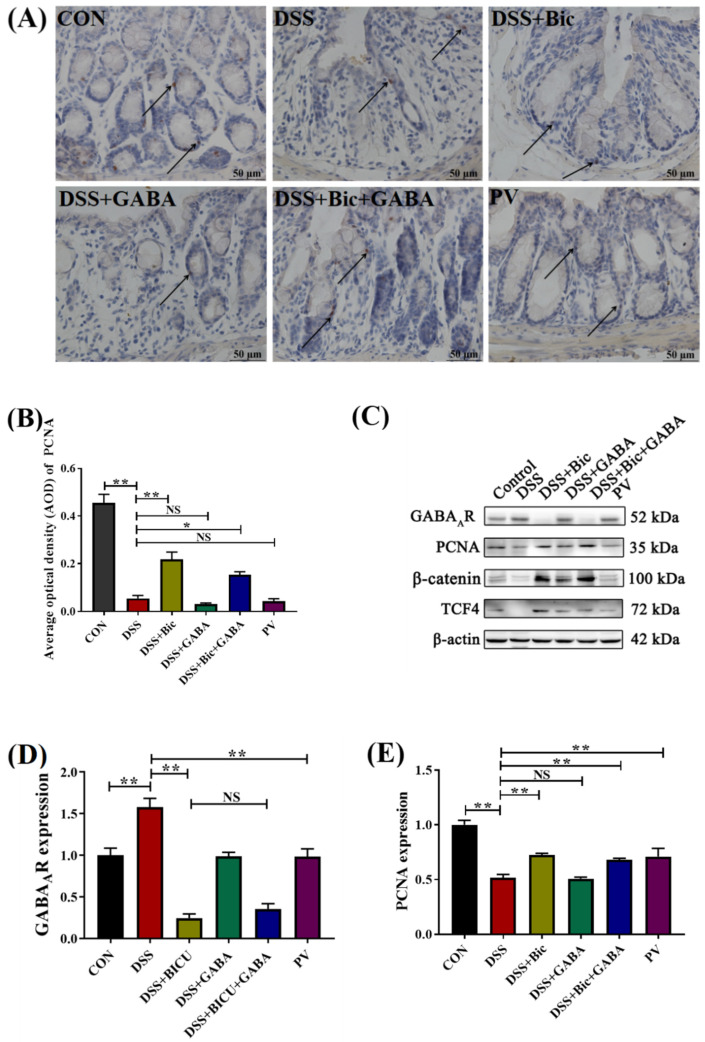

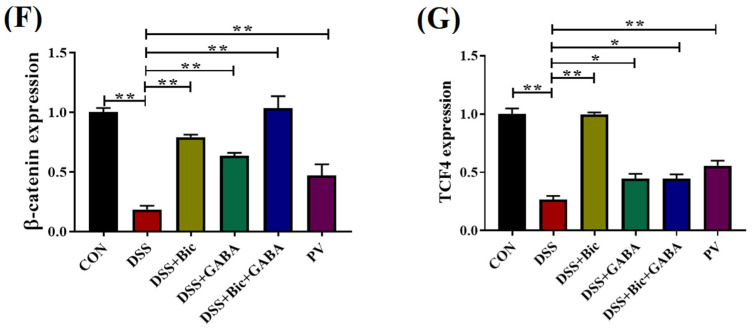
Expression levels of Wnt signaling pathway−associated proteins and PCNA in colon tissues of DSS−induced mice. (**A**) Representative pictures of colon tissue stained by immunohistochemistry with anti−PCNA (brown). Arrows point towards the PCNA signal. (**B**) Statistical analysis of PCNA expression. (**C**) GABA_A_R, PCNA, β−catenin, and TCF4 expression in the mouse colon was determined by Western blot. β−Actin was used as loading control. (**D**–**G**), Quantitative analysis of GABA_A_R, PCNA, β−catenin, and TCF4 protein levels by ImageJ (*n* = 6 per group). Data are presented as mean ± SEM. * *p* < 0.05, ** *p* < 0.01. NS indicates no significant change.

To examine the mechanism whereby inhibition of GABA_A_R is able to promote the proliferation of intestinal cells, the expression levels of Wnt signaling pathway−associated proteins and proliferating cell nuclear antigen (PCNA) in the colon of mice were assessed. PCNA expression could be detected in the nucleus of the crypts and intestinal gland epithelial cells (Figure 4A). The average optical density (AOD) values of the sections showed a significantly decreased cellular proliferation in the DSS and DSS + GABA groups (*p* < 0.05). In comparison to the DSS group, the expression of PCNA was significantly increased in the DSS + Bic and DSS + Bic + GABA groups (*p* < 0.05). The results in Figure 4C–G, suggest that β−catenin and TCF4 were significantly downregulated in the DSS group compared with the control group (*p* < 0.05). It is noteworthy that the protein levels of PCNA, β−catenin, and TCF4 were significantly increased after Bic, Bic + GABA, and *L. casei* Zhang treatment (*p* < 0.05). The protein level of GABA_A_R significantly decreased after treatment with *L. casei* Zhang, which further confirmed the results in Figure 3. Taken together, these results demonstrate that inhibition of GABA_A_R could contribute to maintenance of intestinal integrity in acute colitis by increasing the expression levels of PCNA and Wnt signaling pathway proteins. In addition, *L. casei* Zhang also promoted the renewal and repair of colonic epithelium in mice with colitis.

### 2.5. DSS−Induced Colitis Is Associated with Increased Anxiety−like Behavior

**Figure 5:**
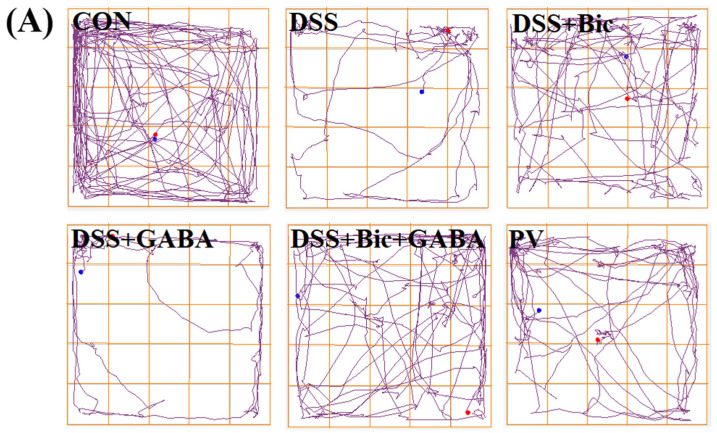

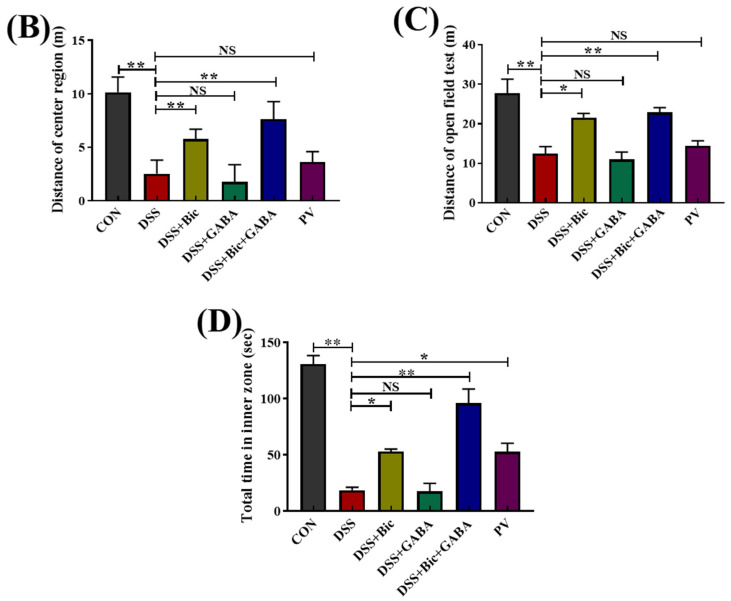
Behavior test of mice in each group. (**A**) Activity tracks from representative mice in an open field during a 5 min recording time. The blue dot represents the start of the track, and the red dot represents the end of the track. (**B**) Distance traveled in the center region. (**C**) Total distance traveled. (**D**) Time spent in the center region. Data are presented as mean ± SEM (*n* = 10 per group). * *p* < 0.05, ** *p* < 0.01. NS indicates no significant change.

In order to determine whether DSS can alter behavior in mice, the open field test (OFT) was used to assess behavior. Our results showed that DSS−induced mice exhibited an increase in anxiety−like behavior, as demonstrated by the shorter distance in the total and center regions (Figure 5). Meanwhile, the results revealed that control mice spent more time exploring than DSS−induced animals in the center of the open field. However, colitis mice treated with Bic exhibited a significantly increased distance in the total and center regions (*p* < 0.05), with increased time spent in the center of the open field. In addition, the time spent in the center region of the PV group was significantly increased compared to the DSS group (*p* < 0.05), and no difference between the DSS group and the DSS + GABA group was found. Taken together, these results suggest that Bic treatment attenuated anxiety−like behavior in mice with colitis.

### 2.6. GABA_A_R Modulates the Gut Microbiota in Mice with Colitis

**Figure 6:**
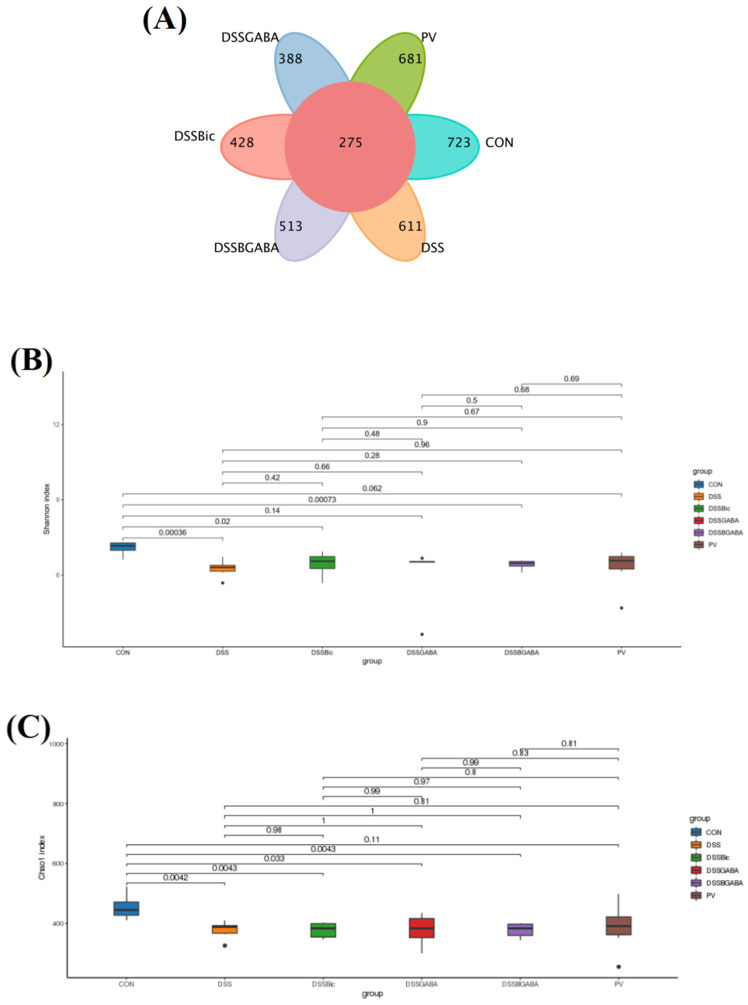

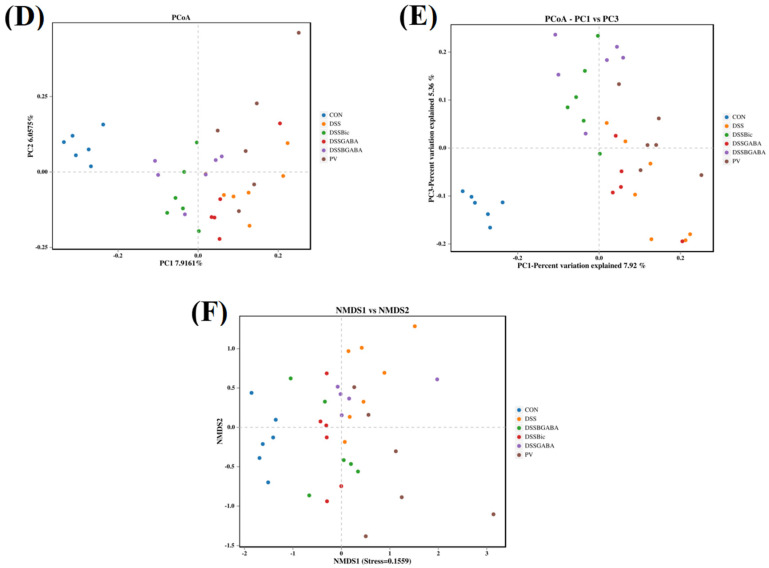
Diversity indexes of microbiota in the mice colon. (**A**) Venn diagram of identified OTUs. (**B**,**C**) α−diversity analyzed by Chao1 and Shannon indices. (**D**–**F**) β−diversity analysis of gut microbiota of each group.

Next, we assessed whether GABA_A_R regulates intestinal microflora. As shown in Figure 6A, the numbers of unique operational taxonomic units (OTUs) in the control, DSS, DSS + Bic, DSS + GABA, DSS + Bic + GABA, and PV groups were 723, 611, 428, 513, 388, and 681, respectively. These six groups shared 275 OTUs among intestinal microbiota. Next, α−diversity was analyzed using the observed species diversity. As shown in Figure 6B,C, the Chao1 and Shannon indices indicated that DSS treatment significantly decreased species richness of bacteria in the gut, and no changes in α−diversity were found between the DSS group and other groups. The overall structure of gut microbiota that was investigated by principal coordinates analysis (PCOA) and non−metric multidimensional scaling (NMDS) analysis showed that the DSS group displayed a shifted clustering of bacterial composition, which was distinct from the control group, suggesting that gut microbiota dysbiosis was induced by DSS treatment (Figure 6D–F). However, Bic treatment remarkably changed the structure of microbiota. Collectively, these results suggest that inhibition of GABA_A_R in the colon could modulate the structure of gut microbiota, partly alleviating the reduction in species richness of the bacterial population upon DSS treatment.

**Figure 7:**
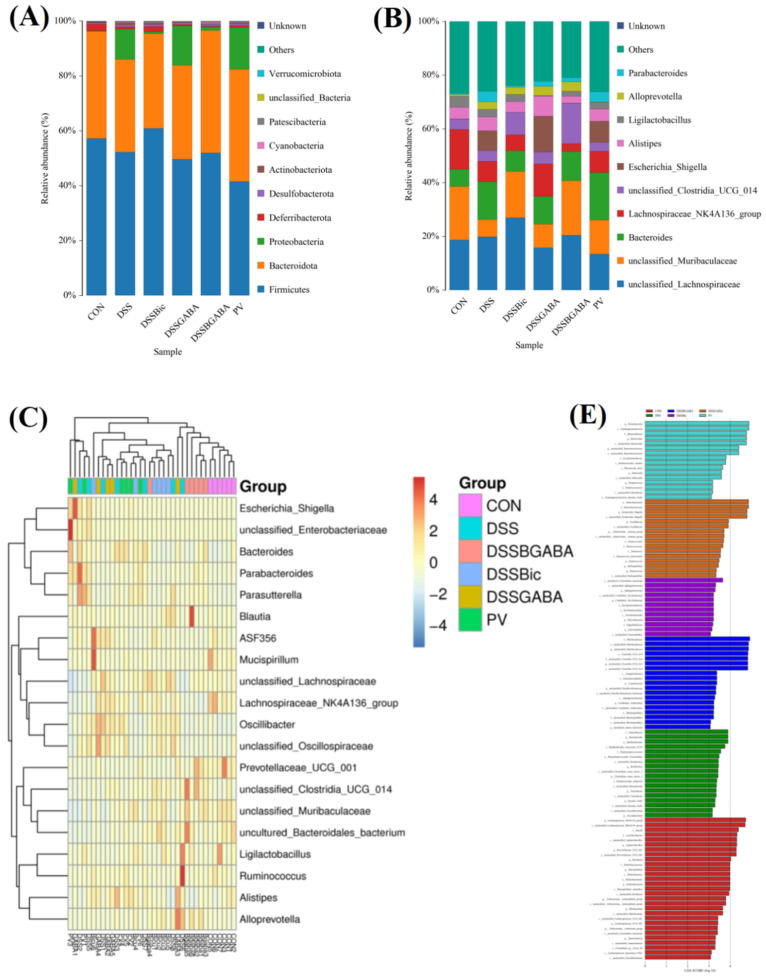

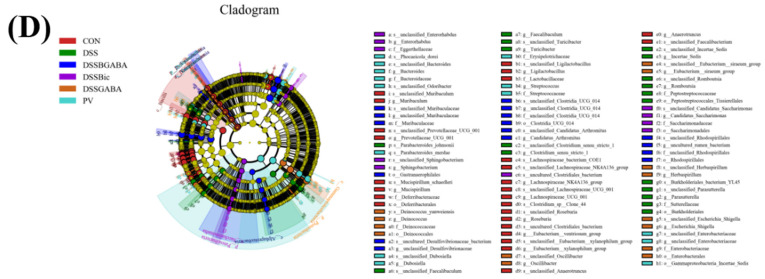
Analysis of the microbial composition. (**A**,**B**) Relative abundances of bacterial taxa at the phylum and genus levels. (**C**) Heatmap of taxa in six groups at the genus level. (**D**,**E**) LEfSe analysis of the dominant biomarker taxa among the six groups.

In order to further study specific differences in the microbiota composition between different groups, we characterized the microbiota community structure profiles of the mice in the six groups. At the phylum level, the bacterial population was primarily composed of Firmicutes, Bacteroidota, Proteobacteria, Deferribacterota, Desulfobacterota, Actinobacteriota, Cyanobacteria, and Patescibacteria (Figure 7A). At the genus level, the DSS group showed an increase in *Bacteroides*, *Escherichia Shigella*, and *Alistipes* and a decrease in Muribaculaceae and *Lachnospiraceae_NK4A136*_group compared with the control group (Figure 7B). Meanwhile, compared to the DSS group, the increased content of beneficial Muribaculaceae in the intestinal microbiota of DSS−treated mice was further elevated by Bic or *L. casei* Zhang. A community heatmap was used to determine the microbial populations at the genus level (Figure 7C). The populations of *Prevotellaceae_UCG_001*, Muribaculaceae, Bacteroidales, and *Ligilactobacillus* were found to be significantly decreased, and the abundance of *Escherichia Shigella*, *Parabacteroides*, and *Parasutterella* was obviously enhanced after DSS or DSS + GABA treatment. Interestingly, Bic or *L. casei* Zhang treatment partly reversed the DSS−induced upregulation of the relative abundance of *Escherichia Shigella*, *Parabacteroides*, and *Parasutterella*. Moreover, we performed a linear discrimination analysis (LDA) using the LDA effect size (LEfSe) algorithm to identify operational microbial taxa that were differentially abundant in each group (Figure 7D,E). After *L. casei* Zhang treatment, relative abundances of Proteobacteria, Bacteroides, and Enterobacteriaceae were significantly increased.

## 3. Discussion

GABA_A_R proteins, which are the major mediators of rapid inhibitory synaptic transmission in the mammalian nervous system, mediate the effects of the neurotransmitter GABA. Recent reports suggested that GABA_A_R could regulate various processes such as learning, memory, and seizure and stress responses [25,26,27]. However, emerging evidence now points to the expression of GABA_A_R in the enteric nervous system, and GABA_A_R plays a key role in the spontaneous contractility of the mouse colon and colonic inflammation caused by psychological stress [11,28]. However, the effect of the GABAergic system on colon epithelial proliferation and renewal is not clear. In this study, we used a DSS−induced mouse model to explore the effect of GABAergic signaling on colonic epithelial proliferation and renewal in mice with colitis. Consistent with previously reported results [29], we also observed increased expression of GABAergic−related molecules in DSS−induced mouse colon tissue, while treatment with GABA aggravated colitis in mice, and application of a GABA_A_R inhibitor attenuated the colitis. However, further research is needed to describe the exact molecular mechanisms by which the GABAergic system affects colitis.

UC can induce a variety of extra−intestinal inflammatory manifestations and complications, which poses a great threat to human health. At present, the main therapeutic strategy for UC is drug therapy, including aminosalicylates, corticosteroids, immunosuppressants, antibiotics, and biologic agents, which have many side effects that limit their therapeutic use [20]. Probiotics are living, non−pathogenic bacteria that are increasingly being used to treat colitis. Probiotics such as *Lactobacillus* and *Bifidobacterium* could maintain remission in adults with UC by building a healthy ecosystem and preventing pathogenic infections [30,31]. In this study, we investigated the effects of *L. casei* Zhang on (i) the expression of GABAergic signal proteins in the colon and (ii) DSS−induced colitis in mice. To detect the potential roles of GABA_A_R and *L. casei* Zhang, colon length, body weight loss, and DAI scores were determined, and a histological analysis was performed to compare DSS, DSS + Bic, DSS + GABA, DSS + Bic + GABA, and PV groups. Our findings showed that Bic partially reversed DSS−induced body weight loss and colon length shortening, and the DAI and histological scores of the colon were increased in DSS−induced mice. These improved clinical parameters were likely due to the maintenance of the intestinal barrier by inducing epithelial cell proliferation. It is worth noting that compared with the DSS group, these clinical parameters did not change significantly after GABA addition, which was probably due to the relatively high expression levels of GABAergic signaling proteins in the colon of DSS−induced mice. Therefore, it was difficult to evaluate the effect of exogenous GABA addition on mice with colitis. To further explore the effect of *L. casei* Zhang on the GABAergic system in the DSS−induced mouse colon, we used immunohistochemistry to detect the expression of GABA_A_R in the colon in each group of mice. We found that oral administration of *L. casei* Zhang significantly decreased the expression of GABA_A_R in colonic tissue of colitis mice, suggesting that the protective effect of *L. casei* Zhang against colitis in mice was probably achieved by inhibiting GABA_A_R.

Compared with the study by Ma X [29], our study further revealed that inhibition of GABA_A_R promoted colonic epithelial cell proliferation, regulated gut microbiota and alleviated anxiety−like behavior in mice with colitis. HE and immunohistochemical staining showed that inhibition of GABA_A_R contributed to maintenance of the integrity of colon epithelium after DSS treatment. Considering this positive impact of GABA_A_R inhibition on colonic epithelium, we hypothesized that the GABAergic signals may play a role in the development of colitis by influencing colonic epithelial proliferation. Wnt/β−catenin signaling is a critical driver of stem cell maintenance and cell specialization, which is essential for maintaining the epithelial proliferative compartment and controlling differentiation [32,33,34]. We showed that inhibition of GABA_A_R significantly increased the expression levels of β−catenin, TCF4, and PCNA, suggesting that inhibition of GABA_A_R may exert protective effects against colitis through the Wnt signaling pathway. Meanwhile, similar to GABA_A_R inhibition, oral administration of *L. casei* Zhang also promoted colon epithelial cell proliferation. The above results demonstrate that *L. casei* Zhang decreased GABA_A_R expression in colonic colitis, so we concluded that *L. casei* Zhang upregulated the Wnt signaling pathway by inhibiting GABA_A_R, thus maintaining the integrity of the colon epithelium. GABA and *L. casei* both have long been shown to be associated with anxiety spectrum disorders. *L. casei* could reduce anxiety symptoms in elderly patients by improving mood disorders [35]. In the control of emotionality, GABA_A_R plays a key role in regulating vigilance, anxiety, and mood [36,37]. Thus, we detected the behavioral changes of mice in each group by the OFT. The OFT is a widely used test to assess anxiety−like behavior in rodents [38,39]. The results showed that inhibition of GABA_A_R or *L. casei* Zhang treatment could alleviate anxiety−like behaviors in DSS−treated mice, suggesting a new treatment method for colitis. Studies have demonstrated that ingestion of *Lactobacillus* regulates emotional behavior and central GABA receptor expression in mice via the vagus nerve [40]. However, *L. casei* could also alleviate stress and anxiety in humans and mice through the regulation of gut immune responses and microbiota composition [41,42]. Our results demonstrated that oral *L. casei* Zhang alleviated anxiety−like behaviors in DSS−treated mice by modulating the gut microbiota. Moreover, inhibition of GABA_A_R also alleviated anxiety−like behaviors in colitis, and our above results have shown that *L. casei* Zhang decreased the expression of GABA_A_R in colitis mice. Therefore, the alleviation of anxiety−like behavior in colitis mice by *L. casei* Zhang is probably the result of both inhibition of GABA_A_R and regulation of intestinal microbiota.

There is increasing evidence that probiotics exert positive effects, not only by reinforcing the intestinal barrier of the host, but also by preventing colonization by pathogenic bacteria and modulating the activity of the intestinal immune system at both the cellular and the molecular level [43,44,45]. *Lactobacillus* species have been reported to exert beneficial effects in the context of intestinal inflammation in experimental mouse models [46]. In addition, recent studies have shown that members of the gut microbiome, such as lactic acid bacteria, are able to produce GABA to regulate mental disorders such as depression and anxiety [47,48]. Our results showed that the diversity of the colonic microbial community decreased and dysbiosis of gut microbiota was induced after DSS treatment. PCOA and NMDS analysis indicated that Bic or *L. casei* Zhang treatment could improve the structure of microbiota after DSS treatment. We found a lower relative abundance of *Pharabacteroides*, *Alistipes*, and Muribaculaceae and a higher relative abundance of *Escherichia Shigella* in the DSS and DSS + GABA groups than in other groups. It has been found that treating mice with *Parabacteroides* and *Alistipes* could induce depressive−like behavior [49,50]. *Alistipes* is a genus of indole−positive bacteria that bind to serotonin receptors, reducing serotonin availability and causing a decrease in pleasant feelings. Meanwhile, *Alistipes* has been reported to express GAD, which metabolizes glutamate into GABA. This may be an important reason for the depressive−like behavior induced by DSS in mice [51]. Furthermore, we discovered that the relative abundance of the genus Muribaculaceae was significantly increased in the *L. casei* Zhang and Bic treatment groups. Heatmap and LEfSe analysis showed that *L. casei* Zhang or Bic treatment increased the numbers of *Bacteroides* and *Ligilactobacillus*, which are beneficial bacteria that maintain intestinal health. A growing number of studies have shown that *Lactobacillus* mitigation of DSS−induced colitis is associated with reduced proinflammatory cytokine levels, increased goblet cells, and secretion of antimicrobial peptides, regulating the ratio of Firmicutes/Bacteroidetes and increasing acetate levels [45,52]. Combined with our results, the protective effects of *L. casei* Zhang on colitis were closely related to the inhibition of GABA_A_R, providing new ideas for the clinical treatment of UC with *L. casei* Zhang.

## 4. Materials and Methods

### 4.1. Bacterial Strain

*L. casei* Zhang is a probiotic strain with good tolerance to acid and bile, which was isolated from the natural fermentation of koumiss (mare’s milk) in the Zhenglanqi herder family in Xilin Gol League, Inner Mongolia [53]. In this experiment, *L. casei* Zhang was fermented and freeze−dried to make a powder, which was dissolved to a concentration of 5 × 10^8^ CFU/mL and gavaged daily (300 μL per day).

### 4.2. Animals

Six− to eight−week−old male C57/BL mice weighing 18−22 g were purchased from Beijing Vital River Laboratory Animal Technology Co., Ltd., Beijing, China. They were acclimatized for 1 week before the experiments and maintained in a room with a controlled temperature (22 ± 1 °C) and a 12/12-h light/dark cycle, with free access to food and water. Acute colitis was induced by administering 4% (*w*/*v*) DSS (molecular weight, 36,000−50,000, Yeasen Biotechnology Co., Ltd. (Shanghai, China)) in drinking water for six days. A total of 60 mice were randomly divided into six groups (*n* = 10 mice per group). Mice in the control group received water only during the six−day experiment. Mice in the DSS group received 4% DSS in drinking water. Mice in the DSS + Bic group received a daily i.p. injection of Bic (HY−N0219, purchased from MedChemExpress, MedChemExpress, NJ, USA) at 0.1 mg/kg once per day for seven days with 4% DSS in drinking water. Mice in the DSS + GABA group received a daily i.p. injection of GABA (03835, purchased from Sigma−Aldrich, St. Louis, MO, USA) at 5 mg/kg once per day for seven days with 4% DSS in drinking water. Mice in the DSS + Bic + GABA group received a daily i.p. injection of Bic at 0.1 mg/kg and GABA at 5 mg/kg once per day for seven days with 4% DSS in drinking water. Mice in the PV group received *L. casei* Zhang by oral gavage for seven days before DSS administration. Mice were monitored daily for weight loss, stool consistency, and fecal bleeding. The changes of DAI were measured using the three criteria mentioned above (Table 1) [54].

### 4.3. Behavioral Test

The OFT is widely used to assess the level of anxiety in mice by providing activity tracks, activity time and exploration habits. The mice were randomly selected and placed in a white OFT chamber (45 cm × 45 cm × 45 cm). The bottom surface of the apparatus was divided into 25 squares (9 cm × 9 cm), which were divided into a surrounding area (16 squares near the walls of the chamber) and a central area (9 squares in the middle). The activities of the mice were recorded by a digital camera set above the apparatus. For each test, each mouse was placed in a corner of the apparatus to acclimatize for 1 min, after which all its activities were recorded for 5 min. The apparatus was cleaned with 75% ethanol before the testing of each animal. The methodology used for the OFT was described previously [55]. The total distance of the mice moving in the open box and the time of staying in the central area within the 5 min was recorded; the sum of these two parameters was used to evaluate the general activity of the mice.

### 4.4. Sample Collection

On day six, the mice were anesthetized with 1% pentobarbital sodium (5 mg/100 g body weight) by i.p. injection and euthanized by neck dislocation. The colon samples and intestinal contents were collected, and the colon lengths were measured. Next, approximately 2 cm long distal colonic intestinal segments were harvested, after phosphate−buffered saline (PBS) cleaning, segments were placed in 4% paraformaldehyde for 24 h for hematoxylin and eosin (H&E) staining, and immunohistochemistry. Furthermore, the remaining colons were rinsed with PBS and frozen in liquid nitrogen and stored at −80 °C until protein expression analysis.

### 4.5. Histological Analysis

For histological examination, colons fixed in 4% paraformaldehyde were removed and embedded in paraffin, sectioned (10 μm thick) and stained with H&E. Histological scores were based on four criteria: number of ulcers, depletion of goblet cells, crypt damage, and inflammatory cell infiltration (Table 2) [56,57].

### 4.6. Immunohistochemistry

Paraffin embedded colon tissue was dewaxed with dimethyl−benzene and rehydrated in gradient ethanol. After antigen heat recovery in citrate buffer, the sections were treated with 1% H_2_O_2_ for 20 min to remove endogenous peroxidase activity and blocked with 5% donkey serum for 30 min. The sections were incubated overnight at 4 °C with polyclonal rabbit anti−GABA_A_Rα3 antibody (12708-1-AP, 1:200, Proteintech Group, Chicago, IL, USA) and monoclonal mouse anti−PCNA antibody (1:300, BM-0104, Boster Biological Technology Co., Ltd., Wuhan, China). Then, these sections were washed three times with PBS for 5 min each time. Next, sections were incubated with a 1:100 dilution of the biotinylated donkey anti−mouse/anti−rabbit IgG (H&L) (715-065-151/711-065-152, 1:100, Jackson Immunoresearch, PA, USA) secondary antibody for 1 h at room temperature. After washing three times with PBS, ABC−peroxidase solution (PK−6102; Vector Laboratories, Burlingame, CA, USA) was added, and allowed to stand for 30 min at room temperature. The sections were incubated in 0.01 M Tris−HCl containing 0.02% 3−3′−diaminobenzidine (D5637; Sigma−Aldrich, Saint Louis, MO, USA) and 0.003% H_2_O_2_ for 5−10 min in the dark to observe immunoreactivity. Finally, the sections were stained with hematoxylin to counterstain the nuclei for 5 s. Three slices were randomly chosen from each group to further select five different regions of each slice. Image J 6.0 software (National Institutes of Health, USA) was used to calculate the average OD of GABA_A_Rα3 and PCNA immunopositive substances in colon.

### 4.7. Western Blotting Analysis

The colon tissue was lysed with RIPA lysis buffer (CW2334S; CWBIO, Beijing, China) containing a protease inhibitor cocktail (CW2200S; CWBIO, Beijing, China), and a bicinchoninic acid (BCA) protein assay kit (CW0014; CWBIO, Beijing, China) was used to quantify the total protein. Subsequently, equal amounts of protein (30 μg per lane) were separated utilizing 10% SDS−PAGE gel (Solarbio, Beijing, China), and then transferred onto PVDF membranes (IPVH00010; Millipore, Danvers, MA, USA). After 1 h of protein blocking with 5% skimmed milk, the membranes were incubated with specific primary antibodies, including rabbit anti−GABA_A_Rα3 (12708-1-AP, 1:1000, Proteintech), mouse anti−PCNA antibody (BM−0104, 1:1000, Boster Biological Technology Co., Ltd.), polyclonal rabbit anti−β−catenin (51067-2-AP, 1:1000, Proteintech), polyclonal rabbit anti−TCF4 (22337-1-AP, 1:1000, Proteintech), and monoclonal mouse anti−β−actin (50201, 1:1000, Kemei Borui Science and Technology, Beijing, China). Later, the membranes were incubated with HRP−conjugated goat anti−rabbit IgG (H&L) (A0208, 1:1000, Beyotime Biotechnology, Shanghai, China) or HRP−conjugated goat anti−mouse IgG (H&L) (A0216, 1:1000, Beyotime Biotechnology) for 1 h at 37 °C. The protein bands were detected by ECL developer and chemiluminescence imager. The protein band intensities were quantified by Image J 6.0 software (National Institutes of Health, USA).

### 4.8. Microbial Sequencing

Fecal genomic DNA was extracted by using a QIAamp DNA stool mini kit (Qiagen). PCR amplification was performed using total DNA and primers targeting the V3−V4 region of the 16S rRNA gene. The amplifications were performed using the following conditions: 95 °C for 5 min, followed by 25 cycles of 95 °C for 30 s, 50 °C for 30 s, and 72 °C for 40 s and a final extension step at 72 °C for 7 min. Next, we constructed the genomic library and conducted high−throughput pyrosequencing of the PCR products using an Illumina MiSeq platform. Finally, the obtained reads of data were processed by cutting and removing the chimeric sequences. According to UCLUST, OTUs were clustered at 97% sequence similarity and classified using the NCBI and RDP reference databases. With QIIME 2.0 software (Knight and Caporaso labs, USA), α−diversity analysis including the Shannon and Chao1 indices and β−diversity analysis including PCOA and NMDS analysis were conducted. The biomarkers of different groups were identified by LEfSe analysis.

### 4.9. Statistical Analysis

GraphPad Prism7 software (GraphPad software, USA) was used for statistical analysis. All data are shown as mean ± SEM. Differences between individual groups were analyzed via one−way ANOVA followed by Tukey’s multiple comparison test. A value of *p* < 0.05 was considered statistically significant.

## 5. Conclusions

In conclusion, oral administration of *L. casei* Zhang could alleviate DSS−induced colitis in mice by inhibiting GABA_A_R. GABAergic signaling plays a vital role in colonic epithelial proliferation and is probably involved in modulating the Wnt/β−catenin signaling pathway and gut dysbacteriosis in the colon. GABAergic signaling may serve as a new drug target to treat IBD.

## Figures and Tables

**Table 1:** Disease activity index.

Score	% Weight Loss	Stool Consistency	Bleeding
0	0	normal stool	no bleeding
1	1~5%	slightly loose stool	few blood−tinged stools
2	5~10%	loose stools	slight blooding
3	10~20%	watery stool	gross blooding
4	>20%	−	blood filling the whole colon

**Table 2:** Histological score.

Score	Ulcers	Depletion of Goblet Cells and Crypt Damage	Inflammatory Cell Infiltration
0	0	normal	no signs of inflammation
1	1	moderate goblet cell loss	low leukocyte infiltration
2	2	high goblet cell loss	moderate leukocyte infiltration
3	3	focal loss of crypts	high leukocyte infiltration
4	>3	extensive fibrosis and diffuse loss of crypts	transmural infiltrations

## Data Availability

All figures and data used to support to this study are induced within this article.
